# Federated learning for medical imaging radiology

**DOI:** 10.1259/bjr.20220890

**Published:** 2023-09-13

**Authors:** Muhammad Habib ur Rehman, Walter Hugo Lopez Pinaya, Parashkev Nachev, James T. Teo, Sebastin Ourselin, M. Jorge Cardoso

**Affiliations:** 1 Division of Biomedical Engineering & Imaging Sciences, King’s College London, London, United Kingdom; 2 The London Medical Imaging and AI Centre for Value Based Healthcare, London, United Kingdom; 3 Institute of Neurology, University College London, London, United Kingdom; 4 King’s College Hospital, NHS Foundation Trust, London, United Kingdom

## Abstract

Federated learning (FL) is gaining wide acceptance across the medical AI domains. FL promises to provide a fairly acceptable clinical-grade accuracy, privacy, and generalisability of machine learning models across multiple institutions. However, the research on FL for medical imaging AI is still in its early stages. This paper presents a review of recent research to outline the difference between state-of-the-art [SOTA] (published literature) and state-of-the-practice [SOTP] (applied research in realistic clinical environments). Furthermore, the review outlines the future research directions considering various factors such as data, learning models, system design, governance, and human-in-loop to translate the SOTA into SOTP and effectively collaborate across multiple institutions.

## Introduction

Radiology is a subject where AI has undeniable potential, and learning models have already been making significant breakthroughs in medical imaging analysis.^1^ These models enhance patient care and make diagnostic procedures from various modalities, such as X-ray, CT, and MRI, more effective.^1^ However, the availability of training data that is sufficiently vast, curated, and representative is crucial to this improvement.^1^ Currently, most healthcare research organisations and clinical settings are limited to a centralised methodology where they only have local data access. For this reason, stakeholders have been developing new approaches to work together on a broad scale without endangering patient privacy to build performant and generalisable AI models.^2^ The only way to train robust AI models that accurately represent the whole human population under study and can go from the lab to the clinical and diagnostic system is through such multi-institutional collaboration.^3^

The idea of federated learning (FL) was first put up as a technological remedy for distributed machine learning that protects user privacy. It has been shown that FL makes it possible for numerous partners to jointly train AI models without transferring data, making it easier to train AI models on a massive amount of data. Like collaborative model training on distributed data, swarm learning (SL) uses a network of nodes to aggregate model weights without a central instance. However, FL and SL have a significant drawback: weight updates must be communicated during training and information about the underlying data may be extrapolated from these weight updates. Therefore, such methods should not be regarded as privacy methods but rather as methods for maintaining data governance. This characteristic severely restricts the applicability of collaborative learning schemes. In addition, the sensitivity of medical imaging data hardens the development of potent AI models for disease diagnosis and clinical integration, and data privacy laws and regulations prohibit the use of medical data in such environments where private data can be extracted.^4^ The main purpose of this review is to enlighten medical imaging AI researchers with the recent developments in FL for medical imaging, articulate some key limitations, and envision a few pertinent research challenges. In this review, we evaluate some FL studies that address privacy, security, heterogeneity, data disparity, aggregation, and missing labels-related issues.

## FL for medical imaging

In an ideal FL environment, all participating medical institutions should collaboratively solve the machine learning problem under the control of a centralised orchestration server in a trusted execution environment. FL provides a framework for hospitals and other healthcare institutions to keep their data local since the model is trained by distributing itself around several cross-institutional medical data centres. The global shared model, which is co-owned by all participating institutions, is maintained by the central server, whereby each institution maintains a local version of its model. The server can assess the quality of local models before aggregating the model updates based on pre-determined criteria to disregard malicious and unfavourable model updates, but, unlike centralised learning (CL), global models keep iterating between aggregation servers and the participating institutions to produce high-quality converged and performant FL models which serve the needs of radiologists and clinicians in realistic diagnostic and clinical environments.

A few surveys and reviews were recently published by researchers on the applicability and adoption of FL for health care.^1,4–11^ Although an early study presents a broader review of FL for medical images;^12^ however, to the best of our knowledge, this is the first focused review on FL techniques for medical imaging applications.

## Adoption and early studies

The majority of the early research examined FL’s applicability to medical imaging and contrasted it with other centralised and distributed learning approaches. In a study^2^ conducted in 2018, the effectiveness of FL algorithms and incremental learning methods was examined when used on patients having multi-institutional multi-modal brain scans for gliomas. The study evaluated FL utilising MRI data from 32 institutions, with an average of less than six individuals per institution. With this dispersed data, the FL models were able to train effectively and showed over 99% consistency when compared to outcomes from full data sharing across institutions. However, using data from 16 and 32 institutions, respectively, the best Institutional Incremental Learning (IIL) and Cyclic Institutional Incremental Learning (CIIL) models demonstrated far more instability, with standard deviations ten times higher than FL models.

In 2019, Wang et al^3^ used two institutional datasets to test FL algorithms on pancreatic segmentation in CT images. Institution 1 submitted 420 portal venous phase abdomen CT images manually tagged for the pancreas to aid in preoperative planning for gastric surgery. Institution 2 submitted 486 contrast-enhanced abdomen CT images of pancreatic patients, which were resampled to isotropic spacing and trimmed to a minimum Hounsfield unit intensity. The global FL model performed well in predicting pancreatic tumours, but it performed somewhat worse when compared to the local models trained at each institution. The authors stated that using server-side-quality enhancement approaches could potentially improve the global model’s performance. In another study, Yi et al^13^ presented improvements to the typical U-Net design for brain tumour segmentation in another investigation. They introduced inception modules and dense blocks to their SU-Net design. Experiments with the Low-Grade Glioma dataset revealed that their proposed SU-Net model outperformed the basic U-Net architecture in both federated and non-federated contexts in terms of Area Under Curve and Dice Similarity Coefficient measures.

Several earlier investigations were made to study the viability of FL for medical imaging applications. Camajori et al^14^ evaluated the latency and model quality trade-offs in synchronous and asynchronous FL contexts using a modified U-Net model and publicly available BraTS and private clinical MRI datasets. According to their findings, large FL models – typically ranging from 30 to 150 MB in every learning round – may clog communication channels in wireless or large-scale FL networks. Similarly, Dou et al^15^ performed a multicentre feasibility study on COVID-19 CXR pictures of 132 patients from seven different hospitals in three different countries. The findings show that FL models are excellent in detecting CT abnormalities in COVID-19 patients and providing scalable and low-cost tools for estimating lesion burden and clinical management.

Similarly, Dayan^16^ used a CXR dataset from 20 global hospitals and trained FL models to predict clinical outcomes (such as the need for mechanical breathing treatment or death within the next 24 h) and achieved 95% sensitivity and 88.2% specificity scores across all hospitals. Lee et al^17^ collected 8457 thyroid ultrasound pictures from six universities and trained various deep learning networks (VGG19, ResNet50, ResNext50, SE-ResNet50, and SE-ResNext50) in FL conditions. Their reported results suggest that the area under the receiving operating characteristic (AUROC) remained between 75.20 and 86.72% in FL conditions, compared to 73.04 to 91.04% for traditional deep learning models. Linardos et al,^18^ on the other hand, used federated transfer learning with a 3D-CNN network pre-trained on an action recognition dataset, then included shape prior knowledge to retrain a cardiac MRI binary classifier. They assessed its performance in terms of leave-centre-out and out-of-site generalisability and discovered good findings.

Because centralisation of FL processes might create delays due to disagreements over control and model ownership, a feasibility study on whole-brain MRI T1 images was performed using BrainTorrent, a peer-to-peer FL system.^19^ The client-specific investigation revealed a 1–4% improvement in Dice score performance when utilising BrainTorrent over centralised FL systems, and BrainTorrent produced more robust aggregated and personalised models. FL also enabled cross-institutional COVID-19 identification studies to benefit from private data. FL implementations of deep neural networks (DNNs) such as COVID-Net, ResNeXt, ResNet18, and MobileNet_v2 outperformed centralised implementations.^20,21^

## Research on FL for medical imaging

This section presents a review of current SOTA research on FL for medical images.

### Partial model sharing

Despite claims of privacy protection and minimal data exposure, experienced attackers can nevertheless acquire access to patients' data. Early investigations on multiparametric preoperative MRI images assessed multimodal and multisegmentation tasks with the goal of minimising soft Dice loss using the Adam optimiser.^22^ Furthermore, privacy risks were reduced by exposing just 10% of the shared model using partial model sharing approaches such as selective parameter sharing and gradient clipping with differential privacy via the sparse vector technique. Similarly, FLOP, a partial model sharing approach, was introduced by keeping the final few layers private from the server while sharing the remainder of the model for federated averaging.^23^ When compared to the traditional FedAvg method, the FLOP’s experimental evaluation with a COVID-19 dataset and six distinct model architectures demonstrated much superior accuracy, privacy guarantees, and personalisation.

### Gradient-level privacy preservation

Model inversion attacks are the most prevalent in FL systems, in which hostile individuals reconstruct images from global models and subsequently target their peers. Gradient-level privacy preservation approaches can aid in the defense against these attacks. The Differentially Private Gradient Descent (DPGD) approach for semantic segmentation in CT images extends Differential Privacy (DP) guarantees to gradient-based optimisation by clipping gradients using the L2 norms of each minibatch.^24^ The optimisation step is then performed after adding Gaussian noise to the averaged minibatch gradients. Despite minor privacy-utility trade-offs, differentially private stochastic gradient descent (DP-SGD) totally defeats privacy-centred attacks, while large-sized models have been shown to be more resilient to model inversion attacks than smaller ones. However, providing strong privacy protections while providing maximum value remains difficult owing to small-sized datasets and the limited number of sites in the hospital networks.

Another interesting solution for preserving gradient-level privacy and preventing model inversion and membership inference attacks by untrusted centres is homomorphically encrypted FL (HEFL). Researchers suggested a HEFL method for multicentric radiology and pathology datasets that outperformed locally and centrally trained models.^25^ Despite strong privacy assurances, HEFL is vulnerable to convergence failure as a result of adversarial weight changes from defective or malicious clients. However, model participants are mainly trustworthy or honest-but-curious people, which reduces this attack vector slightly.

### Heterogeneous datasets

FL systems must primarily deal with heterogeneous datasets that are not distributed independently and identically (non-IID) because of differences in scanning technologies, data collecting procedures, human abilities, data annotation methodologies, and data storage and processing systems. Researchers conducted extensive experiments to learn about the effects of non-IID datasets on FL systems and the effects of data partitions with varying degrees of skewness in quantity and label distributions.^26^ Their suggested weighted average for FedSGD and weighted loss, on the other hand, dramatically reduce the quantity and label distribution skews. Alternatively, during conventional FedAvg, averaging the mean and variance in batch normalisation (BN) across all centres helps to prevent skew-induced BN performance loss.

The non-IIDness of datasets causes activation-divergence even when common classes are present on distinct scanning systems. Researchers developed a prior that increases per-class activation vectors while minimising per-system activation vectors based on the notion of highest entropy.^27^ On short CXR datasets, the activation-divergence with the suggested FedMax technique remained negligible and equal to FedAvg. However, given the substantial variability in large-scale non-IID datasets, the problem may persist. An alternative solution, Federated Disentanglement (FedDis), tackles the non-IID issue by separating the form and appearance of the brain’s anatomical structure in MRI images and then exchanging just the shape parameters for anomaly detection.^28^ FedDis surpasses the best baseline FL techniques by 11%.

### Domain adaptation

Handling systemic data discrepancy in fMRI distributions is another problem when considering scanning system variables. Researchers developed a federated learning method that included Gaussian and Laplacian noises to protect the privacy of shared local model weights, followed by the multisite mixture-of-experts technique for domain adaptation.^29^ Furthermore, the federated adversarial domain alignment approach was used in conjunction with a domain-specific local feature extractor and a global discriminator to enable the generalisation of multisource distant domains into a shared target domain. However, the suggested strategies enhance some but not all multisite fMRI classification. The adversarial domain identifier-based feature alignment approach was utilised to align the intermediate latent space distributions between the source and target locations, as well as to minimise errors around the cranium and lesion regions in *T*_2_-weighted sequences.^30^ Memory-aware curriculum learning on a multisite breast cancer dataset was also employed to improve domain alignment and increase FL classifier performance.^31^ The presence of noisy latent distributions (owing to differential privacy approaches) and the non-IID features of FL datasets, on the other hand, are still major difficulties in creating effective domain adaptation algorithms.

### Variation-awareness

FL gets difficult in the presence of uncommon disorders and image-level differences. FedRare is an intracentre supervised contrastive learning approach for acquiring highly separable latent features, in which the server picks and provides the reliable latent features and the centres jointly compute intraclient contrastive loss.^32^ When tested on a skin lesion segmentation dataset, FedRare beats SOTA algorithms. Similarly, the variation-aware FL (VAFL) architecture decreases image-level fluctuations across centres.^33^ VAFL is built on a privacy-preserving generative adversarial network, notably PPWGAN-GP, which creates synthetic images and then applies a modified CycleGAN for image-to-image translation at each centre before training any classifier. However, VAFL is plagued by visual distortion in malignant areas, which is not a concern for classification tasks but might be devastating with segmentation tasks.

### Image reconstruction

Reconstruction of medical images in FL settings is difficult since images are not acquired in image space and academics have little experience to federated datasets. Researchers used two layers of federated MRI (FedMRI) reconstruction.^34^ First, the server maintains a global encoder that is shared by all centres. Then, since each centre has a unique data distribution, the centre-specific decoders maintain domain-specific features to efficiently reconstruct pictures. A weighted contrastive regularisation approach was also used to ensure optimisation level adjustments in client-server variances and to improve global model convergence. Alternatively, the Federated Learning of Generative IMage Priors (FedGIMP) approach employs unconditional models, which generalise better and are more adaptable to multicentre datasets.^35^ FedGIMP reconstructs pictures in two steps as well, with an unconditional adversarial model initially generating a global image prior to synthesis through latent variables, which is then merged with subject-specific imaging operators for high-quality MRI reconstruction.

### Federated averaging

The traditional federated averaging (FedAvg) approach directly averages the local model weights from all centres and generates new global model weights. However, considering data and system-level heterogeneities, finding a robust aggregating approach is a substantial task. FedCostWAvg is a weighted federated averaging technique presented by researchers that weights local models of 3D-UNet by the amount of local datasets (Federated Tumor Segmentation Challenge) and training gains in respective centres.^36^ FedCostWAvg amplifies more informative updates, therefore it outperforms FedAvg. Fed-CBT, on the other hand, employed a weighted average strategy over graph neural networks to generate a single representative connectivity map from multicentre multiview brain connectomic datasets, but it does not operate well in non-IID situations.^37^ FedFocus was suggested for COVID-19 CXR pictures to pivot the federated learning process by dynamically stabilising the aggregation process depending on training loss, and it beats classical FedAvg^38^ slightly. In addition, for Byzantine-tolerant FL, researchers employed a distance-based outlier suppression approach in which an aggregation server computes the cosine and Euclidean distances between distinct centre updates and assigns outlier scores to each centre.^39^ Finally, the weighted average is calculated using the outlier ratings from each centre. On two medical imaging datasets (CheXpert and HAM10000), its outlier identification effectively protects against model poisoning attacks in both IID and Non-IID FL contexts. HarmoFL is another framework that addresses data heterogeneity at the client and global server levels by applying the amplitude normalisation technique.^40^ HarmoFL beats other SOTA (FedBN, FedProx, and MOON) techniques in breast cancer histology image classification, histology nuclei segmentation, and prostate MRI segmentation tasks.^40^

### Feature-aware aggregation

The majority of aggregation algorithms take server-level settings into account and conduct population-wise aggregations. Considering client-level characteristics while aggregating, on the other hand, becomes advantageous in many circumstances. FedMix is a label-independent adaptive aggregation algorithm that makes good use of labels from pixels, bounding boxes, and images.^41^ FedMix enables discriminative feature representation for all participating centres by employing an adaptive weight assignment technique. When tested on brain tumor and skin lesion segmentation tasks, FedMix greatly beats SOTA approaches. Similarly, Bernecker et al suggested two modality-based feature normalisation algorithms that outperformed SOTA methods on multisite multimodality datasets.^42^ Alternatively, SplitAVG, a heterogeneity-aware FL technique that divides the network into two subnetworks, the server and the institutional subnetwork,^43^ was developed. After calculating the bias from each institutional subnetwork, SplitAVG concatenates intermediate feature maps. SplitAVG’s efficiency in the presence of varied image acquisition, labeling, and quantity skew was proved in an experimental assessment on the BraTs segmentation dataset.

### Unlabelled data or partial annotations

Unlabelled medical images provide a significant difficulty for learning. Self-supervised learning approaches, such as contrastive learning (CL), can efficiently pre-train a neural network from unlabelled data and fine-tune the network to execute downstream tasks with limited annotated data, but the limited variation in centres' data hampers CL algorithm performance in FL environments. To perform segmentation tasks on volumetric medical pictures with little annotations, the Federated Contrastive Learning (FCL) system was proposed.^44^ The framework allows for the interchange of various contrastive features across training networks before performing global structural matching to create well-aligned unified feature spaces for all centres. The suggested FCL framework outperforms SOTA FL approaches in an experimental assessment on a cardiac MRI dataset. Another FCL framework was suggested that employs MoCo as an intranode CL model, and it again performed exceptionally well when the global model attained 90% accuracy with just 3% data labels.^45^ Researchers also proposed Split Learning,^46^ model distillation,^47^ global model optimisation,^48^ ongoing learning,^49^ performance efficiency,^50,51^ personalisation,^52,53^ and a study of COVID-19^54^ in FL settings.

Table 1 presents a comparative overview of notable studies.

**Table 1.** Literature Analysis

Method	Problem	Solution	Strengths	Limitations	Baseline/SOTA
**Partial Model Sharing** ^22^	Model inversion attacks	Selective Parameter Sharing+Differential Privacy	Minimum exposure of patient data	Privacy-Utility trade-off, the more noise the less accurate the models are	Centralised learning
**FLOP** ^23^	Model inversion attacks	Partial Model Sharing	Minimum exposure of patient data	Privacy techniques were not implemented	FedAvg
**DP-SGD** ^24^	Model inversion attacks	Differentially Private Stochastic Gradient Descent	Robust against privacy attacks	Large-batch implementations are hard with DP-SGD	Centralised learning with non-protected gradients
**P2P FL** ^25^	Privacy Attacks	Iterative Continual Learning + Synaptic Intelligence	Robust against privacy attacks	Data heterogeneity impacts precision and sensitivity	Iterative Continual Learning without Synaptic Intelligence
**FedMax** ^27^	Data Heterogeneity causes activation divergence	Maximum Entropy-based Prior	Balances activation vectors across multiple data sources	Larger number of classes may degrade the performance of FedMax	FedAvg
**FedDis** ^28^	Learning from heterogeneous data sources is hard	Federated Disentangled Representation Learning	Mitigates statistical heterogeneity across different scanners	Handling variations in parameter space across different data sources degrades the performance	N/A
**HarmoFL** ^40^	Feature heterogeneity causes domain shifts	Amplitude Normalisation+Weight perturbation	Simultaneously handles both local and global drifts	Need to be tested with large set of model participants	FedBN, FedProx, MOON, FedAdam, FedNova
**FedMix** ^41^	SOTA methods work with standard image annotations across all the data sources	Label-agnostic unified FL framework	Local models use all available labels, server performs adaptive weight assignment across all the data sources	Experiments were made with small dataset	FedAvg, FedST
**SplitAVG** ^43^	Data heterogeneity causes performance drops	Heterogeneity-aware FL using simple network split+feature map concatenation	Effectively overcomes performance drops issues	It only handles statistical heterogeneity	Centralised learning, CWT, FedAvg, SplitNN, FedSGD, FedSGD + GN, FedAvgM, FedAvg + SD
**Domain Adaptation** ^29^	Systemic data differences cause domain shifts across FL training networks	Decentralised iterative optimisation+Domain adaptation	The variations in data sampling and collecting result in sparse datasets	Domain adaptation methods are not always beneficial with FL	Single-site, cross-site, Mix, Ensemble
**FL-MRCM** ^30^	Domain shifts introduce sub-optimality in generalisable model	FL-MR with cross-site modelling	Cross-site modelling aligns the latent spaces between source and target domains	Comparison with SOTA methods is missing	Cross-site, Fused Features, FL-MR
**Memory-aware curriculum FL** ^31^	The frequency and order of sample collection impacts the learning mechanism	Memory-aware curriculum learning+unsupervised domain adaption	Controls the order of training data samples and prioritises the forgotten samples	Coping with Unbalanced and Non-IID data is hard	Fed (Federated) Fed-CL, Fed-Align
**FedIIC** ^32^	Imbalance class distributions introduce bias	Intraclient contrastive learning, interclient contrastive learning	Uniform embedding distribution across all clients	Need to be tested with realistic scenarios	FedAvg, FedProx, MOON, FedProc, FedFocal, FedRS, FedLC, CReFF
**VAFL** ^33^	Cross-client variations results in imbalanced datasets	Privacy-preserving GAN, CycleGAN	Captures variations across the sample space	Variations in manual annotations needs to addressed, Image distortions need to be handled during image-to-image translation	Local Learning, Centralised Learning
**FedMRI** ^34^	Domain shifts degrade the model performance	Specifity-preserving FL for image reconstruction	Benefits in collaborative reconstructions when clients have unique distributions.	Repeated adversarial training between client and server slows down the training process	FedAvg, FedProx, FedBN, FL-MRCM
**FedGIMP** ^35^	Conditional models generalise poorly on non-IID datasets	Cross-site learning of a generative MRI prior+subject-specific injection of the imaging operator	Improves reliability against domain shifts in the imaging operator	Adapting the prior is hard during inference	FL-MRCM, FedGAN, LG-Fed, FedMRI

## State-of-the-Practice, Limitations, and Future Research Directions

**Figure 1.**
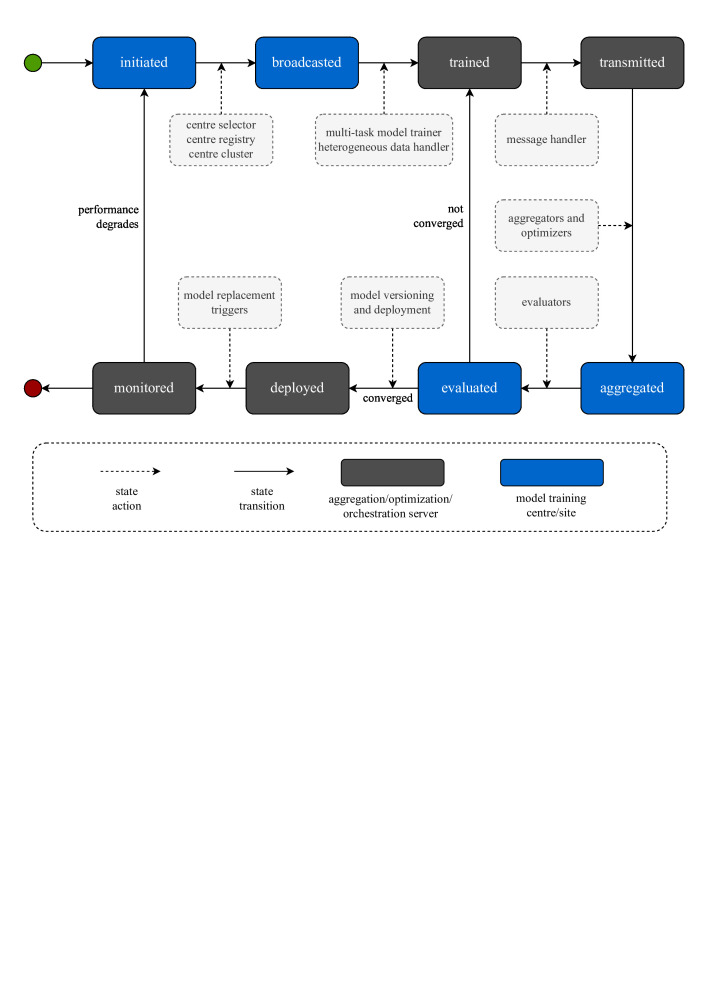
A model’s lifecycle in federated learning environments in realistic clinical settings. (1) the learning process is initiated by an orchestration server, (2) the server selects, registers, and/or clusters participating institutions, (3) the server broadcasts the training and evaluation configurations along with information about datasets, (4) participating institutions execute their local single/multitask trainers and heterogeneous data handlers, (5) participating institutions optionally perform local model enhancements and transmit local models to the server, (6) the server performs aggregation and/optimisation, and/or enhances global model, (7) the server evaluates the global model, (8) the server sends the model for retraining if it does not meet the desired convergence/performance criteria. After the model converges, (9) the server then sends the converged model for versioning and possible deployment at participating institutions, and finally (10) the participating institutions keep monitoring the model performance and either initiate new training cycles or abandon the model if it is not required anymore.

FL is primarily an in-production learning system where FL algorithms may behave significantly different than lab environments. Therefore, most SOTA methods need to be integrated into FL tools, and then re-evaluation should be made to understand their actual utility in state-of-the-practice (SOTP) environments. Ideally, an SOTP is designed to be a fully functioning FL system which is ready to be deployed in realistic hospital environments and which can cater all the basic performance requirements in terms of privacy, security, connectivity, and governance. However, considering the basic FL lifecycle for SOTP environments, as presented in Figure 1, the adoption of FL in multi-institutional collaborative research environments is still in its early phases. Therefore, in addition to solving learning problems, there is a need to define the holistic research agenda considering various facets of FL platforms and medical imaging AI applications. We outline key research directions considering the basic properties of data, learning models, FL systems, governance, and human-in-the-loop.

Although multiple SOTA methods effectively handle data heterogeneity issues, none of the existing methods was tried and tested in SOTP environments and across multiple experiments. Therefore, SOTP frameworks should enable scanner-agnostic and modality-specific federated AI-enabled data acquisition policies to acquire and reconstruct image scans effectively. This approach will help in acquiring high-quality medical images across the varying participating institutions. Similarly, federated, private, and cross-site data exploration can aid in effective data preparation with decreased image distribution shifts. Likewise, federated image annotations on both synthetic and realistic images can marginally increase the data quality by minimising the label-skews and label-scarcity issues across the federated training networks. Since the degrading quality of scanners and misaligned subject-orientations increase noisy and poorly constructed images, federated data preparation and labelling can increase the number of clean images. However, considering differences in time zones while bringing human-in-the-loop, image acquisition protocols, communication networks, subject-and-technician readiness, and availability of expert annotators, the availability of federated datasets is a major challenge.

Unlike SOTA methods, learning from unseen federated datasets is a major challenge in SOTP systems. Ideally, data exploration should be minimised and researchers should not have access to complete datasets. However, researchers should be given access to sufficient data to prepare the correctly functioning FL code which could be submitted to FL platforms to train FL models across collaborating institutions. To this end, conventional data anonymisation techniques should be applied before handing over the patients’ data or synthetic medical images should be generated from actual datasets and it should be made available for medical AI researchers.

The robustness of server-level optimisers and aggregators is desired considering heterogeneity and inaccessibility of remote data, and the various privacy (linkability and utility) and security threats (model-inversion and free-riders attacks). SOTA methods effectively use differential privacy techniques to preserve local and global models. Also, secure multiparty computation and homo-morphic encryption were tested well on multi-institutional datasets. However, the same methods must still be deployed and tested with SOTP methods.

Since SOTA methods mainly focus on the learning part, SOTP FL systems need to consider system-level issues such as the distribution of global models across training networks, synchronising and gathering model updates from participating sites, handling missing model updates, scheduling (re-)training workflows, and ensuring scalable data processing across the training networks. Since FL models are expected to run for a longer period, FL systems need to ensure continuous training, evaluation, testing, integration, deployment, and versioning of learning workflows. Federated experiment definition and federated databases and registries are still needed in SOTP FL systems. In addition, the federated continuous monitoring of training sites and FL workflows is needed for effective model development of SOTP FL environments. Likewise, robust communication protocols that can cater for the seamless transfer of large imaging models are also essential to SOTP FL systems. Considering the economics, human expertise, institutional policies, and involvement of multiple stakeholders (*e.g.,* patients, radiographers, data annotators, clinicians, data engineers, researchers, model developers, DevOps engineers, model owners, and ethics committees), SOTP FL systems should provide end-to-end governance, traceability, and accountability mechanisms.

## Conclusion

The emergence of FL is revolutionising the field of medical imaging, where several SOTA methods are being used to successfully learn from unseen data without jeopardising patients’ privacy. This study reveals that data heterogeneity is the most active research topic, with several local and global model augmentation strategies, as well as some intrasite and cross-site techniques, reported by researchers to address data heterogeneity. Similarly, SOTA methods proposed early techniques for privacy preservation, data augmentation, model distillation, domain adaptation, aggregation and optimisation, performance enhancement, and model personalisation techniques. However, there are still many unanswered problems regarding how to create SOTP FL frameworks given the nature of FL systems and the need for an implementation in actual hospital settings.
